# 3-{4-[(4-Meth­oxy­benzyl­idene)amino]-3-phenyl-5-sulfanyl­idene-4,5-dihydro-1*H*-1,2,4-triazol-1-yl}-1,3-diphenyl­propan-1-one

**DOI:** 10.1107/S160053681103488X

**Published:** 2011-08-31

**Authors:** Wei Wang, Qing-lei Liu, Yan Gao, Xiao-yu Jia, Jing-jing Zhang

**Affiliations:** a School of Perfume and Aroma Technology, Shanghai Institute of Technology, Shanghai 200235, People’s Republic of China; b School of Chemical Engineering, University of Science and Technology LiaoNing, Anshan 114051, People’s Republic of China

## Abstract

In the title mol­ecule, C_31_H_26_N_4_O_2_S, the phenyl ring attached to the 1,2,4-triazole ring forms dihedral angles of 65.4 (2), 63.4 (2) and 62.2 (2)° with the other three rings. The 1,2,4-triazole ring makes dihedral angles of 78.0 (2), 87.9 (2), 24.9 (2) and 62.8 (2)° with three phenyl rings and the methoxyphenyl ring.

## Related literature

For the crystal structures of related 1,2,4-triazole-5(4*H*)-thione derivatives, see: Al-Tamimi *et al.* (2010 ▶); Fun *et al.* (2009 ▶); Gao *et al.* (2011 ▶); Tan *et al.* (2010 ▶); Wang *et al.* (2011 ▶); Zhao *et al.* (2010 ▶).
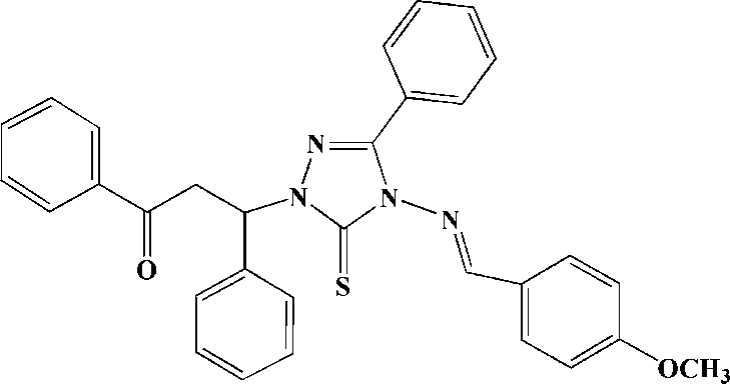


## Experimental

### 

#### Crystal data

C_31_H_26_N_4_O_2_S
*M* *_r_* = 518.62
Monoclinic, 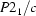

*a* = 6.0131 (12) Å
*b* = 13.940 (2) Å
*c* = 31.490 (4) Å
β = 92.007 (6)°
*V* = 2638.1 (8) Å^3^
*Z* = 4
Mo *K*α radiation
μ = 0.16 mm^−1^
*T* = 113 K
0.20 × 0.12 × 0.10 mm

#### Data collection

Rigaku Saturn CCD area-detector diffractometer
Absorption correction: multi-scan (*CrystalClear*; Rigaku/MSC, 2005 ▶) *T*_min_ = 0.969, *T*_max_ = 0.984
16481 measured reflections
4616 independent reflections
3734 reflections with *I* > 2σ(*I*)
*R*_int_ = 0.053

#### Refinement

*R*[*F* ^2^ > 2σ(*F* ^2^)] = 0.067
*wR*(*F* ^2^) = 0.157
*S* = 1.16
4616 reflections
344 parameters
H-atom parameters constrained
Δρ_max_ = 0.29 e Å^−3^
Δρ_min_ = −0.26 e Å^−3^

### 

Data collection: *CrystalClear* (Rigaku/MSC, 2005 ▶); cell refinement: *CrystalClear*; data reduction: *CrystalClear*; program(s) used to solve structure: *SHELXS97* (Sheldrick, 2008 ▶); program(s) used to refine structure: *SHELXL97* (Sheldrick, 2008 ▶); molecular graphics: *SHELXTL* (Sheldrick, 2008 ▶); software used to prepare material for publication: *SHELXTL*.

## Supplementary Material

Crystal structure: contains datablock(s) global, I. DOI: 10.1107/S160053681103488X/bh2374sup1.cif

Structure factors: contains datablock(s) I. DOI: 10.1107/S160053681103488X/bh2374Isup2.hkl

Supplementary material file. DOI: 10.1107/S160053681103488X/bh2374Isup3.cml

Additional supplementary materials:  crystallographic information; 3D view; checkCIF report

## Figures and Tables

**Fig. 1.**
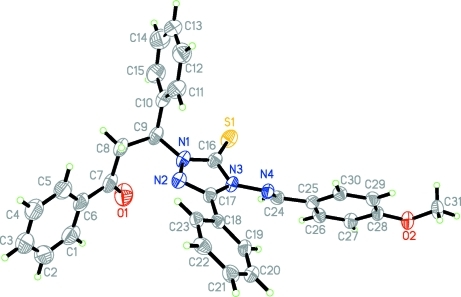
View of the title molecule showing displacement ellipsoids at the 60% probability level.

## supplementary crystallographic information

### Comment

In continuation of structural study of Mannich bases derivatives synthesized by reactions of the amino heterocycles and aromatic aldehydes in our group (Wang *et al.*, 2011), we present here the crystal structure of the title compound (Fig. 1).

The bond lengths and angles in the molecule are found to have normal values comparable with those reported in the related 1,2,4-triazole-5(4*H*)-thione derivatives (Al-Tamimi *et al.*, 2010; Fun *et al.*, 2009; Tan *et al.*, 2010; Wang *et al.*, 2011). The C16 and C17 atoms in the 1,2,4-triazole ring show distorted C*sp*^2^ hybridization states with the bond angles of 101.8 (3)° (N1—C16—N3), 129.5 (2)° (N3—C16—S1), 110.0 (3)° (N2—C17—N3) and 127.3 (3) ° (N2—C17—C18), which are similar to angles reported for other triazole derivatives (Zhao *et al.*, 2010; Gao *et al.*, 2011). There are four phenyl or benzene  rings in this structure. Phenyl ring attached to the 1,2,4-triazole ring forms the dihedral angles of 65.4 (2), 63.4 (2) and 62.2 (2)° with other three rings.

### Experimental

The title compound was synthesized by the reaction of 4-methoxybenzaldehyde (2.0 mmol) and 3-(4-amino-3-phenyl-5-thioxo-4,5-dihydro-1*H*-1,2,4-triazol-1-yl)-1,3-diphenylpropan-1-one (2.0 mmol) in refluxing ethanol. The reaction progress was monitored *via* TLC. The resulting precipitate was filtered off, washed with cold ethanol, dried and purified to give the target product as colorless solid in 81% yield. Crystals suitable for single-crystal X-ray analysis were grown by slow evaporation of a solution in chloroform-ethanol (1:1).

### Refinement

H atoms were positioned geometrically and refined as riding (C—H = 0.95–1.00 Å) and allowed to ride on their parent atoms, with *U*_iso_(H) = 1.2*U*_eq_(parent) or 1.5*U*_eq_(parent).

### Crystal data

**Table:**

C_31_H_26_N_4_O_2_S	*F*(000) = 1088
*M**_r_* = 518.62	*D*_x_ = 1.306 Mg m^−^^3^
Monoclinic, *P*2_1_/*c*	Mo *K*α radiation, λ = 0.71073 Å
Hall symbol: -P 2ybc	Cell parameters from 6676 reflections
*a* = 6.0131 (12) Å	θ = 1.5–28.0°
*b* = 13.940 (2) Å	µ = 0.16 mm^−^^1^
*c* = 31.490 (4) Å	*T* = 113 K
β = 92.007 (6)°	Prism, colourless
*V* = 2638.1 (8) Å^3^	0.20 × 0.12 × 0.10 mm
*Z* = 4	

### Data collection

**Table:**

Rigaku Saturn CCD area-detector diffractometer	4616 independent reflections
Radiation source: rotating anode	3734 reflections with *I* > 2σ(*I*)
multilayer	*R*_int_ = 0.053
Detector resolution: 14.63 pixels mm^-1^	θ_max_ = 25.0°, θ_min_ = 1.3°
φ and ω scans	*h* = −7→7
Absorption correction: multi-scan (*CrystalClear*; Rigaku/MSC, 2005)	*k* = −16→15
*T*_min_ = 0.969, *T*_max_ = 0.984	*l* = −37→37
16481 measured reflections	

### Refinement

**Table:**

Refinement on *F*^2^	Primary atom site location: structure-invariant direct methods
Least-squares matrix: full	Secondary atom site location: difference Fourier map
*R*[*F*^2^ > 2σ(*F*^2^)] = 0.067	Hydrogen site location: inferred from neighbouring sites
*wR*(*F*^2^) = 0.157	H-atom parameters constrained
*S* = 1.16	*w* = 1/[σ^2^(*F*_o_^2^) + (0.0562*P*)^2^ + 1.9562*P*] where *P* = (*F*_o_^2^ + 2*F*_c_^2^)/3
4616 reflections	(Δ/σ)_max_ = 0.001
344 parameters	Δρ_max_ = 0.29 e Å^−^^3^
0 restraints	Δρ_min_ = −0.26 e Å^−^^3^
0 constraints	

### Fractional atomic coordinates and isotropic or equivalent isotropic displacement parameters (Å^2^)

**Table:**

	*x*	*y*	*z*	*U*_iso_*/*U*_eq_	
S1	0.23615 (15)	0.69419 (6)	0.33669 (3)	0.0366 (3)	
O1	0.3196 (5)	0.7950 (2)	0.48255 (9)	0.0547 (8)	
O2	0.1052 (4)	0.15537 (16)	0.24902 (7)	0.0337 (6)	
N1	0.5402 (4)	0.73453 (18)	0.40025 (8)	0.0282 (6)	
N2	0.7135 (4)	0.69513 (18)	0.42388 (8)	0.0271 (6)	
N3	0.5571 (4)	0.58826 (17)	0.38083 (8)	0.0245 (6)	
N4	0.5420 (4)	0.50339 (17)	0.35608 (8)	0.0253 (6)	
C1	0.5395 (7)	0.8105 (3)	0.56280 (13)	0.0468 (10)	
H1	0.3998	0.7786	0.5615	0.056*	
C2	0.6540 (8)	0.8187 (3)	0.60105 (12)	0.0523 (11)	
H2	0.5935	0.7920	0.6259	0.063*	
C3	0.8573 (8)	0.8656 (3)	0.60374 (13)	0.0512 (11)	
H3	0.9359	0.8710	0.6303	0.061*	
C4	0.9454 (7)	0.9043 (3)	0.56763 (12)	0.0482 (10)	
H4	1.0840	0.9371	0.5694	0.058*	
C5	0.8309 (7)	0.8954 (3)	0.52858 (12)	0.0433 (9)	
H5	0.8935	0.9215	0.5038	0.052*	
C6	0.6266 (6)	0.8490 (2)	0.52528 (11)	0.0380 (9)	
C7	0.4977 (6)	0.8366 (2)	0.48405 (12)	0.0375 (9)	
C8	0.5941 (6)	0.8789 (2)	0.44398 (11)	0.0365 (8)	
H8A	0.7566	0.8671	0.4447	0.044*	
H8B	0.5713	0.9492	0.4442	0.044*	
C9	0.4928 (6)	0.8383 (2)	0.40247 (11)	0.0324 (8)	
H9	0.3278	0.8475	0.4024	0.039*	
C10	0.5829 (6)	0.8909 (2)	0.36457 (11)	0.0330 (8)	
C11	0.7910 (6)	0.8697 (3)	0.34881 (11)	0.0387 (9)	
H11	0.8776	0.8190	0.3610	0.046*	
C12	0.8718 (7)	0.9226 (3)	0.31521 (12)	0.0473 (10)	
H12	1.0120	0.9067	0.3041	0.057*	
C13	0.7509 (8)	0.9976 (3)	0.29786 (12)	0.0510 (11)	
H13	0.8080	1.0343	0.2753	0.061*	
C14	0.5448 (9)	1.0187 (3)	0.31375 (14)	0.0624 (13)	
H14	0.4607	1.0707	0.3022	0.075*	
C15	0.4606 (7)	0.9650 (3)	0.34621 (13)	0.0466 (10)	
H15	0.3167	0.9790	0.3561	0.056*	
C16	0.4396 (5)	0.6727 (2)	0.37249 (10)	0.0284 (7)	
C17	0.7243 (5)	0.6060 (2)	0.41084 (9)	0.0244 (7)	
C18	0.8904 (5)	0.5381 (2)	0.42774 (9)	0.0225 (7)	
C19	0.8585 (5)	0.4386 (2)	0.42829 (9)	0.0272 (7)	
H19	0.7240	0.4117	0.4169	0.033*	
C20	1.0228 (6)	0.3795 (2)	0.44547 (10)	0.0306 (8)	
H20	1.0005	0.3121	0.4458	0.037*	
C21	1.2204 (6)	0.4177 (2)	0.46231 (10)	0.0312 (7)	
H21	1.3333	0.3765	0.4737	0.037*	
C22	1.2516 (6)	0.5166 (2)	0.46237 (10)	0.0298 (7)	
H22	1.3856	0.5430	0.4742	0.036*	
C23	1.0891 (5)	0.5767 (2)	0.44532 (9)	0.0261 (7)	
H23	1.1116	0.6441	0.4455	0.031*	
C24	0.3397 (5)	0.4742 (2)	0.34988 (9)	0.0259 (7)	
H24	0.2228	0.5062	0.3637	0.031*	
C25	0.2871 (5)	0.3929 (2)	0.32204 (9)	0.0250 (7)	
C26	0.0753 (5)	0.3513 (2)	0.32370 (10)	0.0284 (7)	
H26	−0.0311	0.3776	0.3420	0.034*	
C27	0.0199 (5)	0.2725 (2)	0.29886 (10)	0.0290 (7)	
H27	−0.1234	0.2443	0.3003	0.035*	
C28	0.1763 (5)	0.2344 (2)	0.27151 (9)	0.0261 (7)	
C29	0.3856 (5)	0.2765 (2)	0.26850 (9)	0.0281 (7)	
H29	0.4902	0.2516	0.2495	0.034*	
C30	0.4381 (5)	0.3550 (2)	0.29371 (9)	0.0256 (7)	
H30	0.5804	0.3839	0.2917	0.031*	
C31	0.2434 (6)	0.1194 (3)	0.21661 (10)	0.0376 (8)	
H31A	0.2660	0.1697	0.1954	0.056*	
H31B	0.1713	0.0639	0.2029	0.056*	
H31C	0.3875	0.1000	0.2293	0.056*	

### Atomic displacement parameters (Å^2^)

**Table:**

	*U*^11^	*U*^22^	*U*^33^	*U*^12^	*U*^13^	*U*^23^
S1	0.0310 (5)	0.0299 (5)	0.0482 (5)	0.0041 (4)	−0.0067 (4)	−0.0010 (4)
O1	0.0489 (17)	0.0576 (18)	0.0587 (17)	−0.0203 (15)	0.0179 (14)	−0.0148 (14)
O2	0.0352 (13)	0.0321 (12)	0.0341 (12)	−0.0010 (10)	0.0021 (11)	−0.0119 (10)
N1	0.0257 (15)	0.0209 (13)	0.0379 (15)	0.0004 (11)	0.0020 (12)	−0.0048 (11)
N2	0.0248 (14)	0.0239 (14)	0.0325 (14)	−0.0004 (11)	0.0007 (12)	−0.0030 (11)
N3	0.0248 (14)	0.0188 (13)	0.0299 (13)	0.0006 (11)	−0.0014 (11)	−0.0048 (10)
N4	0.0298 (15)	0.0211 (13)	0.0250 (13)	0.0005 (11)	−0.0005 (11)	−0.0041 (10)
C1	0.055 (3)	0.031 (2)	0.056 (2)	−0.0015 (18)	0.020 (2)	−0.0044 (17)
C2	0.076 (3)	0.042 (2)	0.040 (2)	0.006 (2)	0.018 (2)	−0.0017 (17)
C3	0.068 (3)	0.036 (2)	0.049 (2)	0.010 (2)	0.003 (2)	−0.0068 (18)
C4	0.055 (3)	0.036 (2)	0.054 (2)	−0.0043 (19)	0.005 (2)	−0.0063 (18)
C5	0.051 (2)	0.035 (2)	0.045 (2)	−0.0052 (18)	0.0121 (18)	−0.0039 (16)
C6	0.045 (2)	0.0234 (17)	0.046 (2)	0.0024 (16)	0.0117 (17)	−0.0082 (15)
C7	0.037 (2)	0.0250 (17)	0.051 (2)	−0.0038 (16)	0.0169 (18)	−0.0121 (15)
C8	0.040 (2)	0.0231 (17)	0.047 (2)	−0.0015 (15)	0.0122 (17)	−0.0096 (15)
C9	0.0267 (18)	0.0220 (16)	0.049 (2)	0.0031 (14)	0.0053 (15)	−0.0078 (15)
C10	0.033 (2)	0.0226 (17)	0.0435 (19)	0.0022 (14)	−0.0023 (16)	−0.0047 (14)
C11	0.034 (2)	0.0339 (19)	0.049 (2)	0.0033 (16)	0.0017 (17)	0.0010 (16)
C12	0.046 (2)	0.050 (2)	0.046 (2)	−0.0097 (19)	0.0047 (19)	−0.0053 (18)
C13	0.078 (3)	0.034 (2)	0.041 (2)	−0.013 (2)	0.004 (2)	0.0018 (17)
C14	0.093 (4)	0.039 (2)	0.055 (3)	0.020 (2)	−0.009 (3)	0.006 (2)
C15	0.051 (2)	0.036 (2)	0.053 (2)	0.0170 (18)	0.0025 (19)	0.0011 (18)
C16	0.0260 (18)	0.0243 (17)	0.0353 (17)	−0.0018 (13)	0.0061 (14)	−0.0017 (13)
C17	0.0236 (17)	0.0225 (16)	0.0273 (15)	−0.0033 (13)	0.0035 (13)	−0.0025 (13)
C18	0.0235 (17)	0.0258 (16)	0.0185 (14)	0.0023 (13)	0.0053 (12)	−0.0026 (12)
C19	0.0283 (18)	0.0259 (17)	0.0273 (16)	−0.0038 (14)	0.0009 (14)	−0.0061 (13)
C20	0.039 (2)	0.0224 (16)	0.0308 (17)	0.0015 (14)	−0.0001 (15)	−0.0023 (13)
C21	0.0325 (19)	0.0339 (18)	0.0269 (16)	0.0067 (15)	−0.0014 (14)	−0.0015 (14)
C22	0.0259 (17)	0.0385 (19)	0.0250 (16)	−0.0036 (15)	−0.0011 (14)	0.0014 (14)
C23	0.0311 (18)	0.0244 (16)	0.0230 (15)	−0.0018 (14)	0.0053 (13)	−0.0032 (13)
C24	0.0258 (18)	0.0262 (16)	0.0257 (15)	0.0004 (14)	0.0024 (13)	0.0006 (13)
C25	0.0260 (18)	0.0240 (16)	0.0252 (15)	0.0018 (13)	0.0011 (13)	0.0003 (13)
C26	0.0279 (18)	0.0312 (18)	0.0263 (16)	−0.0005 (14)	0.0031 (14)	−0.0023 (13)
C27	0.0251 (18)	0.0285 (17)	0.0335 (17)	−0.0034 (14)	0.0025 (14)	−0.0036 (14)
C28	0.0311 (19)	0.0233 (16)	0.0238 (15)	0.0003 (14)	−0.0026 (14)	−0.0038 (12)
C29	0.0296 (18)	0.0298 (17)	0.0252 (16)	0.0041 (14)	0.0033 (14)	−0.0006 (13)
C30	0.0230 (17)	0.0270 (17)	0.0272 (15)	0.0002 (13)	0.0050 (13)	0.0010 (13)
C31	0.042 (2)	0.038 (2)	0.0325 (17)	0.0055 (16)	−0.0014 (16)	−0.0146 (15)

### Geometric parameters (Å, °)

**Table:**

S1—C16	1.662 (3)	C12—C13	1.376 (6)
O1—C7	1.218 (4)	C12—H12	0.9500
O2—C28	1.370 (4)	C13—C14	1.384 (6)
O2—C31	1.429 (4)	C13—H13	0.9500
N1—C16	1.355 (4)	C14—C15	1.378 (6)
N1—N2	1.374 (4)	C14—H14	0.9500
N1—C9	1.477 (4)	C15—H15	0.9500
N2—C17	1.310 (4)	C17—C18	1.463 (4)
N3—C17	1.378 (4)	C18—C19	1.401 (4)
N3—C16	1.393 (4)	C18—C23	1.406 (4)
N3—N4	1.418 (3)	C19—C20	1.382 (4)
N4—C24	1.291 (4)	C19—H19	0.9500
C1—C2	1.371 (6)	C20—C21	1.390 (5)
C1—C6	1.415 (5)	C20—H20	0.9500
C1—H1	0.9500	C21—C22	1.392 (5)
C2—C3	1.386 (6)	C21—H21	0.9500
C2—H2	0.9500	C22—C23	1.381 (4)
C3—C4	1.381 (5)	C22—H22	0.9500
C3—H3	0.9500	C23—H23	0.9500
C4—C5	1.394 (5)	C24—C25	1.461 (4)
C4—H4	0.9500	C24—H24	0.9500
C5—C6	1.389 (5)	C25—C30	1.399 (4)
C5—H5	0.9500	C25—C26	1.402 (4)
C6—C7	1.498 (5)	C26—C27	1.383 (4)
C7—C8	1.526 (5)	C26—H26	0.9500
C8—C9	1.531 (5)	C27—C28	1.402 (4)
C8—H8A	0.9900	C27—H27	0.9500
C8—H8B	0.9900	C28—C29	1.395 (4)
C9—C10	1.517 (5)	C29—C30	1.382 (4)
C9—H9	1.0000	C29—H29	0.9500
C10—C15	1.383 (5)	C30—H30	0.9500
C10—C11	1.394 (5)	C31—H31A	0.9800
C11—C12	1.391 (5)	C31—H31B	0.9800
C11—H11	0.9500	C31—H31C	0.9800
			
C28—O2—C31	118.2 (3)	C13—C14—H14	119.7
C16—N1—N2	114.1 (2)	C14—C15—C10	120.8 (4)
C16—N1—C9	124.8 (3)	C14—C15—H15	119.6
N2—N1—C9	120.7 (2)	C10—C15—H15	119.6
C17—N2—N1	104.7 (2)	N1—C16—N3	101.8 (3)
C17—N3—C16	109.3 (2)	N1—C16—S1	128.7 (2)
C17—N3—N4	123.9 (2)	N3—C16—S1	129.5 (2)
C16—N3—N4	125.4 (2)	N2—C17—N3	110.0 (3)
C24—N4—N3	112.8 (2)	N2—C17—C18	122.7 (3)
C2—C1—C6	120.9 (4)	N3—C17—C18	127.3 (3)
C2—C1—H1	119.6	C19—C18—C23	119.2 (3)
C6—C1—H1	119.6	C19—C18—C17	123.6 (3)
C1—C2—C3	120.5 (4)	C23—C18—C17	117.1 (3)
C1—C2—H2	119.8	C20—C19—C18	119.9 (3)
C3—C2—H2	119.8	C20—C19—H19	120.0
C4—C3—C2	119.8 (4)	C18—C19—H19	120.0
C4—C3—H3	120.1	C19—C20—C21	120.8 (3)
C2—C3—H3	120.1	C19—C20—H20	119.6
C3—C4—C5	120.0 (4)	C21—C20—H20	119.6
C3—C4—H4	120.0	C20—C21—C22	119.5 (3)
C5—C4—H4	120.0	C20—C21—H21	120.2
C6—C5—C4	121.0 (4)	C22—C21—H21	120.2
C6—C5—H5	119.5	C23—C22—C21	120.4 (3)
C4—C5—H5	119.5	C23—C22—H22	119.8
C5—C6—C1	117.8 (4)	C21—C22—H22	119.8
C5—C6—C7	123.2 (3)	C22—C23—C18	120.1 (3)
C1—C6—C7	119.0 (3)	C22—C23—H23	120.0
O1—C7—C6	121.1 (3)	C18—C23—H23	120.0
O1—C7—C8	120.7 (3)	N4—C24—C25	121.2 (3)
C6—C7—C8	118.2 (3)	N4—C24—H24	119.4
C7—C8—C9	114.3 (3)	C25—C24—H24	119.4
C7—C8—H8A	108.7	C30—C25—C26	118.6 (3)
C9—C8—H8A	108.7	C30—C25—C24	123.0 (3)
C7—C8—H8B	108.7	C26—C25—C24	118.4 (3)
C9—C8—H8B	108.7	C27—C26—C25	120.6 (3)
H8A—C8—H8B	107.6	C27—C26—H26	119.7
N1—C9—C10	111.2 (3)	C25—C26—H26	119.7
N1—C9—C8	109.3 (3)	C26—C27—C28	119.7 (3)
C10—C9—C8	110.5 (3)	C26—C27—H27	120.2
N1—C9—H9	108.6	C28—C27—H27	120.2
C10—C9—H9	108.6	O2—C28—C29	124.7 (3)
C8—C9—H9	108.6	O2—C28—C27	114.8 (3)
C15—C10—C11	118.8 (3)	C29—C28—C27	120.6 (3)
C15—C10—C9	119.4 (3)	C30—C29—C28	118.9 (3)
C11—C10—C9	121.8 (3)	C30—C29—H29	120.6
C12—C11—C10	120.0 (3)	C28—C29—H29	120.6
C12—C11—H11	120.0	C29—C30—C25	121.7 (3)
C10—C11—H11	120.0	C29—C30—H30	119.2
C13—C12—C11	120.8 (4)	C25—C30—H30	119.2
C13—C12—H12	119.6	O2—C31—H31A	109.5
C11—C12—H12	119.6	O2—C31—H31B	109.5
C12—C13—C14	119.0 (4)	H31A—C31—H31B	109.5
C12—C13—H13	120.5	O2—C31—H31C	109.5
C14—C13—H13	120.5	H31A—C31—H31C	109.5
C15—C14—C13	120.7 (4)	H31B—C31—H31C	109.5
C15—C14—H14	119.7		
			
C16—N1—N2—C17	0.1 (3)	C9—N1—C16—S1	−3.3 (5)
C9—N1—N2—C17	−172.9 (3)	C17—N3—C16—N1	−3.1 (3)
C17—N3—N4—C24	142.3 (3)	N4—N3—C16—N1	−169.9 (3)
C16—N3—N4—C24	−52.7 (4)	C17—N3—C16—S1	174.8 (2)
C6—C1—C2—C3	0.5 (6)	N4—N3—C16—S1	7.9 (5)
C1—C2—C3—C4	0.0 (6)	N1—N2—C17—N3	−2.2 (3)
C2—C3—C4—C5	−0.7 (6)	N1—N2—C17—C18	179.3 (3)
C3—C4—C5—C6	0.9 (6)	C16—N3—C17—N2	3.4 (3)
C4—C5—C6—C1	−0.4 (5)	N4—N3—C17—N2	170.6 (3)
C4—C5—C6—C7	−179.4 (3)	C16—N3—C17—C18	−178.1 (3)
C2—C1—C6—C5	−0.3 (5)	N4—N3—C17—C18	−11.0 (4)
C2—C1—C6—C7	178.8 (3)	N2—C17—C18—C19	153.5 (3)
C5—C6—C7—O1	179.6 (3)	N3—C17—C18—C19	−24.8 (5)
C1—C6—C7—O1	0.6 (5)	N2—C17—C18—C23	−24.6 (4)
C5—C6—C7—C8	−1.2 (5)	N3—C17—C18—C23	157.1 (3)
C1—C6—C7—C8	179.8 (3)	C23—C18—C19—C20	−0.9 (4)
O1—C7—C8—C9	−18.3 (5)	C17—C18—C19—C20	−179.0 (3)
C6—C7—C8—C9	162.5 (3)	C18—C19—C20—C21	0.0 (5)
C16—N1—C9—C10	−71.5 (4)	C19—C20—C21—C22	0.9 (5)
N2—N1—C9—C10	100.8 (3)	C20—C21—C22—C23	−0.9 (5)
C16—N1—C9—C8	166.2 (3)	C21—C22—C23—C18	0.0 (4)
N2—N1—C9—C8	−21.5 (4)	C19—C18—C23—C22	0.9 (4)
C7—C8—C9—N1	−62.6 (4)	C17—C18—C23—C22	179.1 (3)
C7—C8—C9—C10	174.7 (3)	N3—N4—C24—C25	174.5 (3)
N1—C9—C10—C15	141.4 (3)	N4—C24—C25—C30	−14.1 (5)
C8—C9—C10—C15	−97.0 (4)	N4—C24—C25—C26	166.2 (3)
N1—C9—C10—C11	−41.5 (4)	C30—C25—C26—C27	2.1 (5)
C8—C9—C10—C11	80.0 (4)	C24—C25—C26—C27	−178.1 (3)
C15—C10—C11—C12	0.0 (5)	C25—C26—C27—C28	−0.6 (5)
C9—C10—C11—C12	−177.1 (3)	C31—O2—C28—C29	−7.6 (4)
C10—C11—C12—C13	1.6 (6)	C31—O2—C28—C27	172.3 (3)
C11—C12—C13—C14	−1.3 (6)	C26—C27—C28—O2	178.8 (3)
C12—C13—C14—C15	−0.6 (6)	C26—C27—C28—C29	−1.3 (5)
C13—C14—C15—C10	2.1 (6)	O2—C28—C29—C30	−178.6 (3)
C11—C10—C15—C14	−1.8 (6)	C27—C28—C29—C30	1.6 (5)
C9—C10—C15—C14	175.3 (4)	C28—C29—C30—C25	0.1 (5)
N2—N1—C16—N3	1.8 (3)	C26—C25—C30—C29	−1.9 (5)
C9—N1—C16—N3	174.5 (3)	C24—C25—C30—C29	178.4 (3)
N2—N1—C16—S1	−176.0 (2)		
